# Novel frame-shift mutation in *PKP2* associated with arrhythmogenic right ventricular cardiomyopathy: a case report

**DOI:** 10.1186/s12881-015-0263-1

**Published:** 2015-12-23

**Authors:** Teresa Trenkwalder, Isabel Deisenhofer, Martin Hadamitzky, Heribert Schunkert, Wibke Reinhard

**Affiliations:** Klinik für Herz- und Kreislauferkrankungen, Deutsches Herzzentrum München, Technische Universität München, Lazarettstr. 36, 80636 Munich, Germany; Deutsches Zentrum für Herz- und Kreislaufforschung (DZHK) e.V., Partner Site Munich Heart Alliance, Munich, Germany; Klinik für Radiologie und Nuklearmedizin, Deutsches Herzzentrum München, Technische Universität München, Munich, Germany

**Keywords:** Arrhythmogenic right ventricular cardiomyopathy, PKP2, genetic testing

## Abstract

**Background:**

Arrhythmogenic Right Ventricular Cardiomyopathy (ARVC) is an inherited disease mainly found in young people causing malignant arrhythmias which can result in sudden cardiac death. Due to unspecific symptoms the diagnosis of ARVC is still challenging and requires clinical testing and expert knowledge. Genetic testing of index patients is helpful in the primary diagnosis and further testing of family members may allow for prevention of sudden cardiac death.

**Case presentation:**

We report a case of newly diagnosed ARVC where genetic testing identified a novel familial frame-shift mutation in the *PKP2* gene. Screening of the family members identified both children and the father as mutation carriers following an autosomal-dominant inheritance pattern.

**Conclusion:**

Our findings emphasize the importance of genetic family screening after the identification of a causative mutation in an index case.

## Background

Arrhythmogenic Right Ventricular Cardiomyopathy (ARVC) is a hereditary cardiac disease that predominantly affects the right and possibly the left ventricle and is pathologically characterized by progressive fibrofatty replacement of myocardium [1]. Clinically cardiac rhythm, i.e., palpitations, syncope and sudden cardiac death (SCD), or cardiac function, i.e., right- or bi-ventricular heart failure resembling dilated cardiomyopathy, may be affected. The diagnosis is based on the 2010 Task Force criteria including characteristic electrocardiographic, arrhythmic, structural and/or histological abnormalities [2]. The prevalence is estimated to be 1:1000 to 1:5000 with a mean age at diagnosis of 31 years (±13; range 4–64 years) [3]. ARVC is acknowledged to be a leading cause of ventricular arrhythmias and SCD in young, often athletic subjects below the age of 35 years [1, 2]. As much as 30 – 50 % of ARVC patients have a positive family history and ARVC most commonly follows an autosomal-dominant inheritance. However, incomplete penetrance and highly variable and age-dependent disease expression are observed [1].

## Case presentation

**Fig. 1:**
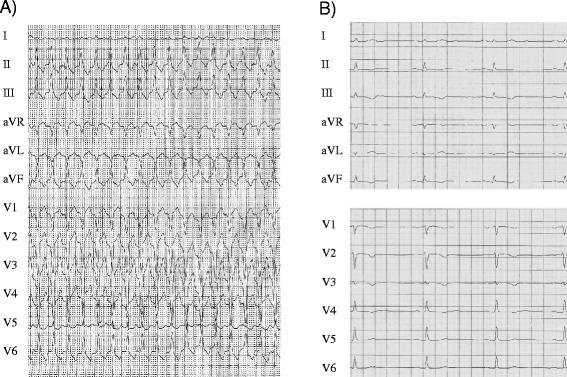
**a** 12-Lead ECG of the index patient (paper speed 25 mm/s) showing ventricular tachycardia with left bundle branch block morphology and inferior axis. **b** Resting 12-Lead ECG upon admission to our hospital showing sinus rhythm (paper speed 50 mm/s). T-wave inversion is present in V1-V4

A 43-year-old female presented with shortness of breath, chest discomfort and light-headedness while cross-country skiing. She was able to call the rescue service herself and upon arrival they detected a wide QRS complex tachycardia with left bundle branch block morphology and inferior axis at 190 bpm that was terminated by administration of 50 mg Ajmalin i.v. (Fig. 1a). In stable condition the patient was transferred to our emergency room for further evaluation. Medical history included repeated episodes of similar symptoms upon physical exertion, yet no syncope. The family history was remarkable of a survived sudden cardiac arrest of the patient’s father at the age of 86 years and recurrent syncopes during exercise of the patient’s daughter.

**Fig. 2:**
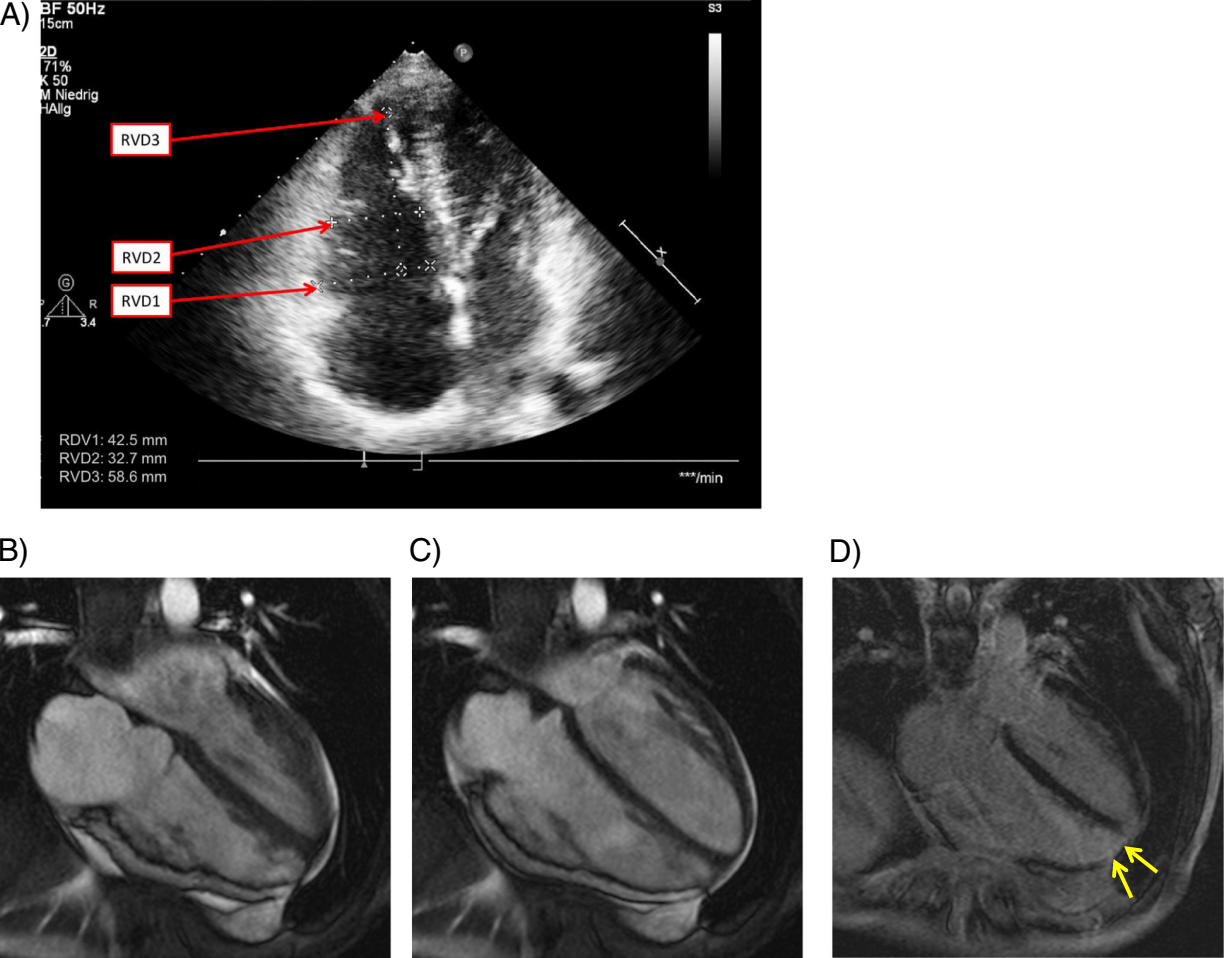
**a** Echocardiography of index patient shows dilatation of the right ventricle. RVD1 (basal right ventricle): 42.5 mm (Mean value (95 % CI) 33 (31–35 mm)). RVD2 (mid right ventricle) 32.7 mm (Mean value (95 % CI) 28 (23–33 mm)). RVD3 (base to apex length) 58.6 mm (Mean value (95 % CI) 71 (67–75 mm)). Mean values (95 % CI) according to Rudski et al. [9]. **b** Cardiac MRI of the index patient reveals moderate enlargement of the right ventricle with an enddiastolic volume of 186 ml (standard value female 140 ± 37 ml) and (**c**) endsystolic volume of 135 ml (standard value female 52 ± 22 ml) resulting in a highly reduced ejection fraction of 27 % (standard value female 64 ± 7 %) Furthermore, the apical posterior wall of the right ventricle showed akinesia and (**d**) late gadolinium enhancement identified apical fibrosis (yellow arrows) in the 4-chamber view. Standard values according to Hudsmith et al. [10]

The resting 12-lead ECG displayed T-wave inversion in leads V1-4 in the absence of bundle branch block configuration (Fig. 1b). Laboratory analysis showed mildly elevated troponin T. Echocardiography showed normal size and function of the left ventricle. However, the right ventricle (RV) was significantly enlarged with reduced function (Fig. 2a). Cardiac MRI displayed a reduced RV ejection fraction of 27 % with moderate RV enlargement and increased RV end-diastolic volume (128 ml) (Fig. 2b, c), as well as akinesia of the apical RV posterior wall with late enhancement in this area (Fig. 2d). The coronary angiogram was normal. 12 lead-Holter monitoring revealed numerous premature ectopic ventricular beats (>7000) and three episodes of sustained ventricular tachycardia around 200 bpm the longest lasting over 20 min with left bundle branch block configuration originating from the RV outflow tract (RVOT). In the electrophysiological study, 3D electroanatomic voltage mapping showed low voltage areas in the apical and basal inferior RV as well as the lateral and medial RV outflow tract. Isoproterenol infusion as well as programmed ventricular stimulation induced the clinically documented ventricular tachycardia (VT) as well as 3 other forms of RV-VT. Radiofrequency energy ablation guided by voltage and activation mapping successfully eliminated all induced VTs and reached non-inducibility of ventricular arrhythmias. Taken together the definite clinical diagnosis of late-onset arrhythmogenic right ventricular cardiomyopathy (ARVC) was made according to the modified Task Force criteria by Marcus et al. [2]. The patient fulfilled two major criteria of ARVC (MRI pathology, repolarization abnormalities) and one minor criterion (arrhythmia).

**Fig. 3:**
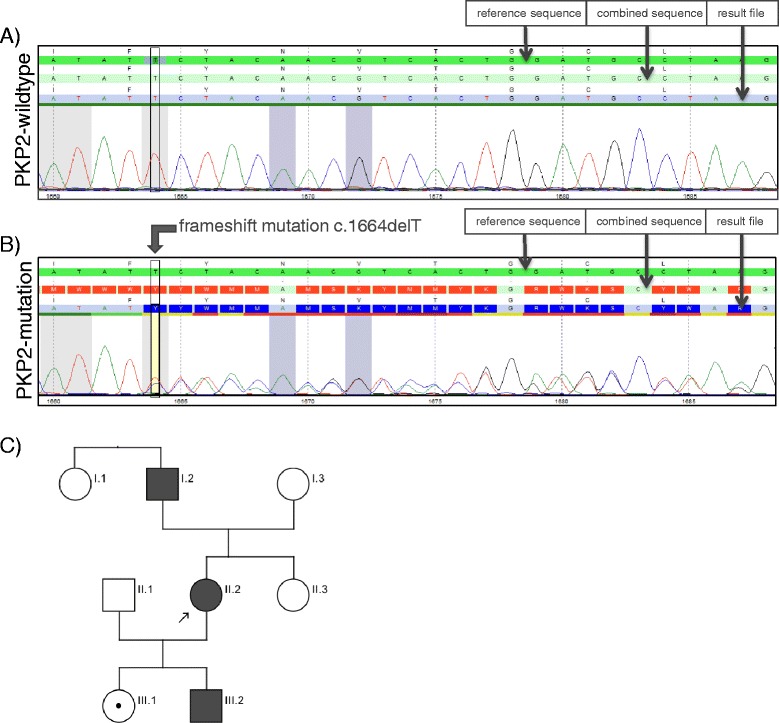
Electropherogram curves and sequences of (**a**) the wildtype sequence and (**b**) the index patient: The index patient shows the frameshift mutation c.1664delT (p.F555Sfs*8) in the PKP2-gene. **c** Pedigree of the index patient (II.2) displaying an autosomal dominant inheritance pattern. Her father (I.2) is also diagnosed with ARVC and both children (III.1 and III.2) are mutation carriers, the son (III.2) fulfills the 2010 Task Force criteria of ARVC and is diagnosed with the disease

During the following days ventricular tachycardia could neither be recorded in repeated Holter monitoring nor induced by controlled exercise stress testing. Nevertheless, we recommended the implantation of an automated implantable cardiac defibrillator (ICD) for secondary prophylaxis of sudden cardiac death, which was initially refused by the patient. The patient agreed, however, to conduct genetic testing of common ARVC genes for eventual substantiation of the diagnosis. While DNA exon sequencing of the ARVC genes desmoglein 2 (DSG2), desmoplakin (DSP), ryanodine receptor 2 (RYR2), transmembrane protein 43 (TMEM43), desmocollin 2 (DSC2), junction plakoglobin (JUP) and transforming growth factor beta 3 (TGFB3) showed no pathological findings, sequencing of the plakophilin-2 (PKP2) gene identified a new variant in exon 7 (performed at the Synlab, Mannheim, Germany). This heterozygous c1664*del*T deletion mutation has not yet been described in the literature (HGMD Professional 2014.1). However, a pathogenic effect of this mutation is very likely, since the frame-shift p.F555Sfs*8 prematurely terminates translation which probably leads to degradation of RNA or composition of an altered protein (Fig. 3a, b). Both mechanisms would result in loss of function of the mutated allele. Confronted with this result, the patient agreed to the ICD implantation and a single-chamber ICD was successfully implanted. In the 6-months follow-up no sustained or non-sustained ventricular tachycardia was recorded in the ICD memory.

Predictive genetic testing of first-degree family members of the index patient for the new *PKP2* mutation disclosed the familial heterozygous mutation in both children (ages 13 and 16) and the father of the patient. Neither her mother, nor her sister and her aunt were carriers of the mutation (Fig. 3c, Table 1). All carriers of the mutation underwent a comprehensive cardiological work-up including 12-lead ECG, exercise stress test, Holter monitoring, echocardiography and MRI of the heart (performed at Deutsches Herzzentrum München, Universitätsklinikum München-Großhadern, Germany).

**Table 1:**
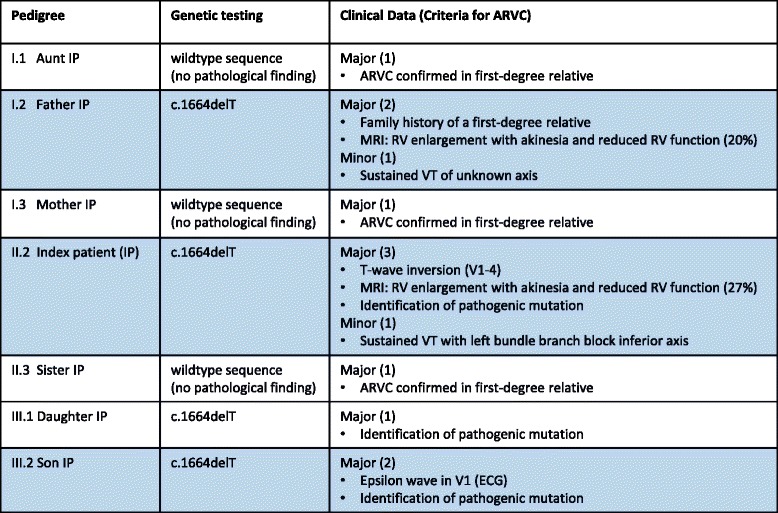
Results of genetic testing and clinical criteria (major, minor) for the diagnosis of ARVC of the index patient and the tested family members. According to the 2010 Task Force criteria diagnosis of ARVC can be made by presence of the following criteria: 2 major criteria or 1 major?+?2 minor criteria or 4 minor criteria (from different categories) [2]. The criteria for diagnosis were fulfilled in the index patient (II.2), her father (I.2) and her son (III.2), all marked in blue. *RV* right ventricle; in brackets number of criteria present

The daughter showed an orthostatic reaction after stress testing resulting in dizziness and pre-syncopy but no arrhythmias were detected during this episode. At present, the remaining cardiological examinations were normal. The son presented with an epsilon potential in lead V1 in the resting ECG. The results of all other tests showed currently no abnormal findings. Nevertheless, with two major criteria being present, ARVC was diagnosed in the son. Both children were recommended to refrain from excessive physical exercise and to perform semi-annual cardiologic check-ups. The 91-year old father of the index patient, currently still in good health, had a long-standing medical history of cardiac diseases, including sick sinus syndrome, implantation of a biologic aortic valve at the age of 81 and one episode of ventricular tachycardia that was successfully terminated with i.v. amiodarone at the age of 86, when he declined the implantation of an ICD. Echocardiography showed significant dilatation of the right ventricle with severely reduced function (TAPSE = 7 mm). Upon MRI the right ventricular ejection fraction was severely reduced (20 %) with akinesia and fibrosis of the lateral wall. Therefore, the diagnosis of ARVC was made.

## Discussion

We report a case of a 43-year old female with newly diagnosed ARVC in whom genetic testing identified a novel frame-shift mutation in the *PKP2* gene. Although the clinical diagnosis was established according to the 2010 Task Force criteria, the patient was reluctant to undergo ICD implantation, since she had not suffered from severe symptoms and felt that the intervention posed a substantial change to her daily life. In our case genetic testing was able to substantiate the diagnosis by the finding of a new frame-shift mutation in the *PKP2* gene and to convince the patient of the need for the ICD implantation. Moreover, a comprehensive family screening detected the mutation in both of her children and her father following an autosomal-dominant inheritance. The disease was also present in an advanced state in the father, and diagnosed in an early asymptomatic stage in the son, whereas the daughter had currently normal test results. While no sensible medical recommendations arose for the nonagenarian father, the teenage children of the patient are now confronted with the fact that they are asymptomatic disease and mutation carriers respectively. As a result, lifestyle modifications such as restraint from exhausting physical activity and cardiologic check-ups on a regular basis are warranted.

### Genetic testing in ARVC

According to the Heart Rhythm Society/European Heart Rhythm Association’s Expert Consensus Statement genetic testing in ARVC can be useful for patients satisfying the 2010 Task Force diagnostic criteria and may be considered for patients with possible ARVC (1 major or 2 minor criteria), while it is not recommended for patients with only a single minor criterion. Mutation-specific genetic testing is recommended for family members after the identification of a causative mutation in an index case [4]. To date, mutations in eight genes are known to cause the disease (*PKP2, DSG2, DSP, DSC2, RYR2, TGFB3, JUP, TMEM43*), with six of them encoding for proteins with importance for desmosomal structure or function *(PKP2, DSG2, DSP, DSC2, JUP, TMEM43)* [5]. Mutations in *PKP2* are most common ranging from 10 – 78 % in ARVC patients [6, 7]. The overall yield of a current generation genetic test for a patient with the clinical diagnosis of ARVC approximates 50 % [7]. However, the frequency of rare variants in ARVC susceptibility genes in healthy volunteers, the so-called background rate of genetic variability, is estimated with 16 %, meaning that 1 in 6 healthy individuals would meet current criteria for a positive ARVC genetic test result [7]. Understanding the genetic variability including the importance of the genetic background mutation rate is important for the interpretation of a genetic test result. For comparison, in long-QT syndrome the rate of “mutations” without clinical phenotype in the three most frequently involved genes *KCNQ1*, *KCNH2*, and *SCN5A* is only 5 % [8]. Ideally, patients should be referred to specialized centers to perform the genetic test and test results have to be interpreted in light of clinical test results. Moreover, mutation characteristics seem to play a role in determining the probability of pathogenicity. Kapplinger et al. [7] found that radical mutations in ARVC susceptibility genes, such as in-frame and frame-shift insertions and deletions, splice junction, and nonsense mutations, are strongly associated with the disease (42.9 % in ARVC cases vs. 0.47 % in healthy controls, *p* < 9.8 × 10^−44^), whereas rare missense mutations are less likely to be related to ARVC and should be interpreted in the context of race and ethnicity, mutation location, and sequence conservation [7]. Radical mutations, as the frame-shift mutation in our patient, are found in approximately ¾ of mutation-positive ARVC cases [7]. Despite the deleterious nature of this novel frame-shift mutation on the gene product the age of diagnosis in our patient and that of her father in particular is high. One can speculate whether this specific mutation goes along with a late-onset of symptoms and is comparatively “benign”, or whether other factors, such as genetic background and external influences (e.g., physical activity) affect the course of the disease. However, it is known that the presentation of ARVC can be highly variable even within families, which is of particular importance for the patient’s clinically asymptomatic children with positive genetic testing in whom preventive measures should be taken. In general our case suggests that genetic evaluation in ARVC can be helpful to establish the definite diagnosis and to influence clinical decision making, but should be viewed as one component of comprehensive clinical assessment and never replace clinical judgment.

## Conclusion

We report a case of newly diagnosed ARVC where genetic testing identified a novel familial frame-shift mutation in the *PKP2* gene. Although not meant to override clinical judgment, the case shows that in index cases genetic testing can be helpful to establish the definite diagnosis and to guide treatment considerations. Among family members mutation-specific genetic cascade screening has diagnostic, prognostic and therapeutic implications. Interpretation of test results for ARVC is challenging and referring patients to specialty centers is strongly encouraged.

## Ethics statement and consent

Written informed consent was obtained from the patient and all family members for publication of this case report and any accompanying images. A copy of the written consent is available for review by the Editor of this journal.

## Abbrevations

ARVC: Arrhythmogenic Right Ventricular Cardiomyopathy (ARVC)
DSC2: desmocollin 2
DSG2: desmoglein 2
DSP: desmoplakin
JUP: junction plakoglobin
PKP-2: plakophilin-2
RYR2: ryanodine receptor 2
TAPSE: tricuspid annular plane systolic excursion
TGFB3: transforming growth factor beta 3
TMEM43: transmembrane protein 43
